# Effects of synbiotics supplementation on anthropometric and lipid profile parameters: Finding from an umbrella meta-analysis

**DOI:** 10.3389/fnut.2023.1121541

**Published:** 2023-02-23

**Authors:** Vali Musazadeh, Maryam Mohammadi Anilou, Mahdi Vajdi, Arash Karimi, Sana Sedgh Ahrabi, Parvin Dehghan

**Affiliations:** ^1^Student Research Committee, Faculty of Nutrition and Food Science, Tabriz University of Medical Sciences, Tabriz, Iran; ^2^Department of Emergency Medicine, Shahid Beheshti University of Medical Sciences, Tehran, Iran; ^3^Student Research Committee, Isfahan University of Medical Sciences, Isfahan, Iran; ^4^Department of Community Nutrition, School of Nutrition and Food Sciences, Isfahan University of Medical Sciences, Isfahan, Iran; ^5^Nutrition Research Center, Department of Clinical Nutrition, School of Nutrition and Food Sciences, Tabriz University of Medical Sciences, Tabriz, Iran; ^6^Nutrition Research Center, Faculty of Nutrition and Food Science, Tabriz University of Medical Sciences, Tabriz, Iran

**Keywords:** synbiotic, lipid profile, anthropometric indices, obesity, meta-analysis

## Abstract

**Introduction:**

Several systematic reviews and meta-analyses have been carried out to assess the impact of synbiotics on lipid profiles and anthropometric parameters. In this regard, an umbrella meta-analysis was performed to provide a more accurate view of the overall impacts of synbiotic supplementation on lipid profile and anthropometric parameters.

**Methods:**

Databases such as PubMed, Scopus, Embase, Web of Science, and Google Scholar were searched for this study from inception to January 2022. A random-effects model was applied to evaluate the effects of synbiotic supplementation on lipid profile and anthropometric parameters. The methodological quality of eligible articles was evaluated using the AMSTAR2 questionnaire. The GRADE approach was used to evaluate the overall certainty of the evidence in the meta-analyses.

**Results:**

Meta-analyses of 17 studies revealed significant decreases in body mass index (BMI) (ES: −0.13 kg/m2; 95% CI: −0.19, −0.06, *p* < 0.001, I^2^ = 0.0%, *p* = 0.870), BW (ES: −1.30 kg; 95% CI: −2.19, −0.41, *p* = 0.004, I^2^ = 88.9%, *p* < 0.001), waist circumference (WC) (ES: −1.80 cm; 95% CI: −3.26, −0.34, *p* = 0.016, I^2^ = 94.1%, *p* < 0.001), low-density lipoprotein cholesterol (LDL-C) (ES: −2.81 mg/dl; 95% CI: −3.90, −1.72, *p* < 0.001, I^2^ = 95.1%, *p* < 0.001), total cholesterol (TC) (ES = −2.24 mg/dl; 95% CI: −3.18, −1.30, *p* < 0.001, I^2^ = 94.5%, *p* < 0.001), and triglyceride (TG) (ES: −0.43 mg/dl; 95% CI: −0.79, −0.07, *p* = 0.019, I^2^ = 78.0%, *p* < 0.001) but not high-density lipoprotein cholesterol (HDL-C) (ES: 0.23 mg/dl; 95% CI: −0.11, 0.56, *p* = 0.193, I^2^ = 45.2%, *p* = 0.051) following synbiotic supplementation.

**Discussion:**

The present umbrella meta-analysis suggests synbiotic supplementation can slightly improve lipid profile and anthropometric indices and might be a therapeutic option for obesity and its related disorders.

**Systematic review registration:**

www.crd.york.ac.uk/prospero, identifier CRD42022304376.

## Introduction

It is established that obesity is a multi-factorial, chronic, treatable, and neurobehavioral condition in which excess body fat mass results in adipose tissue dysfunction and abnormal physical forces of fat mass that lead to a variety of metabolic diseases (1). The increasing outbreak of obesity is one of the most important health concerns worldwide since being overweight increases the risk of several diseases, in particular, hyperlipidemia, diabetes, hypertension, cardiovascular disease (CVD), and cancer (2). Lifestyle interacts with local environmental and genetic factors to diversify the prevalence of obesity among populations.

Several studies have revealed that overweight and obesity by 2030 in women and men will reach 85 and 89%, respectively (1). This increases the risk of obesity-related risks such as coronary heart disease (CHD) by 97%, cancer by 61%, and types 2 diabetes by 21% (2, 3). Dyslipidemia is defined by one or more abnormal lipid concentrations in serum lipids [total cholesterol (TC), low-density lipoprotein cholesterol (LDL-C), triglyceride (TG), and high-density lipoprotein cholesterol (HDL-C)] (4). This problem can be caused by hereditary factors, but in most cases, obesity and overweight lead to this condition. The pathophysiology of typical dyslipidemia apperceived, in obesity is multi-factorial and includes, reduction of circulating TG lipolysis, hepatic overproduction of very-low-density lipoprotein (VLDL), disorder peripheral free fatty acids (FFA) trapping, enhanced FFA fluxes from adipocytes to the liver and other tissues, and the constitution of small dense LDL-C (5, 6). In addition, disruption of the ASP/C3 adesArg pathway may contribute to typical dyslipidemia. Therefore, the management and prevention of dyslipidemia have been considered in recent decades (7). The aim of treatment should be to increase physical activity and improve dietary habits by reducing total calorie intake and decreasing saturated fatty acid (SFA) consumption. Currently, several treatment options target each aspect of dyslipidemia but recent guidelines recommend complex therapies to treat the multiple lipid abnormalities (8–10). In recent years, it is proved that gut dysbiosis (imbalance between pathogenic and beneficial gut microbiome) is linked with diabetes, metabolic syndrome (MetS), dyslipidemia, and obesity by extra energy production altering the metabolism of energy in host and pro-inflammatory signals (11–13). Therefore, balancing the gut microbial flora plays a significant role in human health (14, 15). Synbiotics are nutritional supplements that are a mixture of probiotics and prebiotics in a synergic form (16). Synbiotics include both substrates and advantageous microorganisms, which might have synergic effects on the intestinal tract (17). Synbiotics have a beneficial effect on the host by improving survival and increasing the dose of live microbes in the gastrointestinal tract (18, 19). Numerous studies have indicated that the use of synbiotics could improve the glycemic status, lipid metabolism, markers of liver enzymes, inflammatory mediators, and the function of intestinal microbiota. However, there are differences between studies examining the effect of synbiotics on weight loss (17, 20, 21). Meta-analyses have examined the therapeutic impacts of synbiotics on lipid profile (20, 22) and obesity indices (23, 24); nevertheless, the findings are still inconsistent (25–28). Thus, the current umbrella meta-analysis study was designed to assess the effects of supplementation with synbiotics on TC, HDL-C, TG, and LDL-C levels and anthropometric indices, including body mass index (BMI), body weight (BW), and waist circumference (WC).

## Materials and methods

The research protocol has been registered on PROSPERO (registration number: CRD42022304376). We conducted the current investigation in accordance with the PRISMA (Preferred Reporting Items for Systematic Reviews and Meta-Analyses) (29).

### The search strategy of literature

International scientific databases, such as Web of Science, Embase, PubMed, Scopus, and Google Scholar, were searched for relevant published papers till January 2022. The search technique for MeSH and the keywords utilized in this research are as follows: [(“Synbiotics” OR “Symbiotics” OR “Synbiotic”) AND (lipids OR High density lipoprotein cholesterol OR HDL-C OR Total cholesterol OR TC OR “Low density lipoprotein cholesterol” OR “LDL-C” OR Triglyceride OR TG OR “bodyweight” OR “body weight changes” OR “body mass index” OR “weight loss” OR “obesity” OR OR “BMI” OR “waist circumference” OR “WC”) AND (“meta-analysis” OR “systematic review”)]. The “*” keyword was used to improve the sensitivity of our study methodology. Also, to prevent the loss of research, a thorough search of references to relevant studies was conducted.

### Inclusion and exclusion criteria

We followed these PICO criteria: Population/Patients (P: adults aged 18>), Intervention (I: treated with synbiotic), Comparison (C: control group), Outcome (O: anthropometric and lipid profile parameters), and Study design (S: meta-analysis). The previous meta-analyses, reviewed in the present study, investigated the effects of synbiotic supplementation on anthropometric and lipid profile parameters by using their effect size (ES) values and their corresponding confidence intervals (CI). In addition, other typologies of research studies including *in vivo, in vitro*, and *ex vivo* studies, observational studies, case reports, controlled clinical trials, and quasi-experimental studies were excluded from the present study.

### Assessment of outcomes

The outcomes that were investigated in this umbrella meta-analysis included the effects of synbiotic supplementation on anthropometric indices and lipid profile. Among the anthropometric indices, BMI, BW, and WC were investigated and regarding the lipid profile, four indices of LDL-c, HDL-c, TG, and TC were investigated.

### Data extraction and study selection

According to the qualifying requirements, two distinct reviewers evaluated the articles (SSA and MMA). We began by reviewing abstracts and titles. To establish if a research was appropriate for a meta-analysis, the entire texts of relevant papers were analyzed. Disputes were resolved by reaching an agreement with the senior reviewer (MV). The following information was retrieved from the chosen papers: the names of the first authors, the sample size, the publication year, dose of synbiotics, gender, health condition, length of the intervention, and ESs and their CIs.

### Quality assessment

Two reviewers (VM and SSA) independently assessed the methodological quality of the qualifying articles using the AMSTAR2 questionnaire (30). The questionnaire has 16 questions to which reviewers must respond “Yes” or “Partial Yes” or “No” or “No Meta-analysis.” Critically poor quality, low quality, moderate quality, and excellent quality were assigned to the AMSTAR2 checklist. The third reviewer (MV) was also accountable for settling any conflicts.

### Synthesis of data and statistical analysis

To estimate the pooled effect size, reported effect sizes ESs and CIs were used. The *I*^2^ statistic and Cochrane’s Q test were used to detect heterogeneity. When the *I*^2^ value >50% or the *Q*-test had *p* < 0.1, we considered between-study heterogeneity significant. When considerable heterogeneity existed across studies, we adopted the random-effects model. Subgroup analyses were conducted to identify potential sources of heterogeneity according to a number of variables, including dosage of synbiotic, type of ESs, duration of intervention, and the sample size, age of participants, health condition, bacteria strain type. A sensitivity analysis was performed to detect whether the total ES was associated with a single study. Egger (31) and Begg’s (32) tests were used to determine the small-study effect. Visual analysis of the funnel plot revealed publication bias (33). If publication bias was obvious, trim and fill methods are done. Version 16.0 of STATA was used for all statistical analyses (Stata Corporation, College Station, TX). When *p* < 0.05, values were deemed statistically significant.

### Quality of evidence

GRADE (Standards for the Development, Evaluation, and Evaluation Working Group) criteria were used to evaluate the overall certainty of the evidence in the meta-analyses. The quality of the evidence was categorized according to four assessment criteria: high, moderate, low, and very low (34).

## Results

### Study selection and study characteristics

**FIGURE 1:**
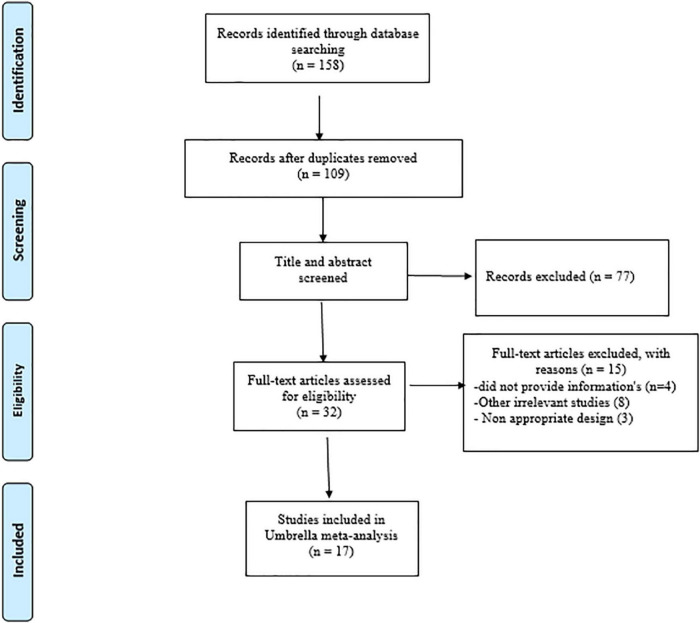
PRISMA flow diagram of selection studies.

A total of 158 papers were found after a thorough search of internet databases. After removing 49 duplicate papers, the titles and abstracts of 109 papers were thoroughly examined, with 32 papers being chosen for full-text evaluation. Finally, 17 papers matched our inclusion criteria and were qualified for the umbrella meta-analyses. A flow chart of the PRISMA study and a study trend is given in Figure 1. The ES measures for studied variables are eight for weight, 11 for BMI, seven for WC, 12 for TG, 11 for TC, LDL-C, and HDL-C. In addition, seven studies were performed in Iran (20, 23, 25, 28, 35–37), four in the USA (38–41), three in China (26, 42, 43), two in Brazil (27, 44), and one in Spain (45). A total of 124 articles involving 7,772 participants were included in the present umbrella meta-analysis. Included studies were performed between 2014 and 2022. The number of subjects in studies ranged between 168 and 2,629. The participants’ average age ranged between 27 and 53 years. In the studies, the intervention lasted between 8 and 20 weeks. Administered synbiotics dosages ranged between 3.4 × 10^8^ and 1.3 × 10^10^ CFU. The quality assessment process was performed in almost all meta-analyses included in the present study, except for one study (25), which did not report the quality assessment. Except for two studies by Hadi et al. (23) and Brasserie et al. (27), which utilized the Jadad score and CONSORT-based checklist, respectively, others applied Cochrane Collaboration’s tool to perform the quality assessment. Table 1 shows the characteristics of the studies that were included.

**TABLE 1:** Study characteristics of included studie.

Reference	No of studies in meta-analysis	Location duration	No of participants in meta-analysis	Age (year)	Intervention	Quality assessment scale and outcome
Cozzolino et al. (45)	3	Spain 11 wk	305	30	Lactobacillus, bifidobacterium, bacillus, streptococcus	Yes (cochrane) 2/3 high
Liu et al. (42)	8	China 18 wk	415	46	Lactobacillus, bifidobacterium, streptococcus	Yes (cochrane) 8/8 high
Suzumura et al. (44)	4	Brazil 17 wk	301	52	Lactobacillus, bifidobacterium, streptococcus	Yes (cochrane) 4/4 high
Hadi et al. (25)	23	Iran 13 wk	1,357	49	Synbiotic food, synbiotic capsule	Yes (cochrane) 22/23 high
Hadi et al. (52)	7	Iran 17.5 wk	419	49.7	Lactobacillus, bifidobacterium	Yes (jadad score) 6/7 high
John et al. (38)	3	USA 8 wk	82	NR	NR	Yes (cochrane) 3/4 high
Sharpton et al. (41)	12	USA 18 wk	769	NR	Different	Yes (cochrane) 8/12 high
Loman et al. (40)	7	USA 16 wk	399	47	Different	Yes (cochrane) 7/7 high
Miao et al. (43)	7	China 11 wk	486	NR	Different	Yes (cochrane) 5/7 high
Hadi et al. (23)	23	Iran 12 wk	1,338	50	Lactobacillus, bifidobacterium, streptococcus	Yes (cochrane) 23/23 high
Heshmati et al. (37)	3	Iran 12 wk	219	27	NR	Yes (cochrane) 3/3 high
Khan et al. (39)	3	USA 20 wk	332	44	Lactobacillus, bifidobacterium, streptococcus	Yes (cochrane) 1/3 high
Li et al. (58)	3	China 10.5 wk	191	NR	Lactobacillus, bifidobacterium, streptococcus, bacillus	Yes (cochrane) 3/3 high
Tabrizi et al. (28)	7	Iran 8 wk	482	51	Synbiotic foods and capsules	Yes (cochrane) 7/7 high
Hadi et al. (36)	4	Iran 10 wk	206	28	Lactobacillus, bacillus, bifidobacterium	Yes (cochrane) 4/4 high
Arabi et al. (35)	5	Iran 15 wk	323	53	Lactobacillus, bifidobacterium, streptococcus	Yes (cochrane) 4/5 (high)

### Evaluation of methodological quality

The methodological quality assessment details using the AMSTAR2 checklist are outlined in Table 2. Three meta-analyses out of 17 meta-analyses included high quality and 14 articles of moderate quality.

**TABLE 2:** Results of assess the methodological quality of meta-analysis.

Study	Q1^1^	Q2	Q3	Q4	Q5	Q6	Q7	Q8	Q9	Q10	Q11	Q12	Q13	Q14	Q15	Q16	Quality assessment
Hadi et al. (20)	Yes	Partial yes	No	Partial yes	Yes	Yes	No	Yes	Yes	No	Yes	Yes	Yes	Yes	Yes	Yes	Moderate
Cozzolino et al. (45)	Yes	Yes	Yes	Yes	Yes	Yes	Yes	Yes	Yes	No	Yes	Yes	No	No	No	Yes	Moderate
Suzumura et al. (44)	Yes	Yes	No	Yes	Yes	Yes	Yes	Yes	Yes	No	Yes	Yes	Yes	Yes	Yes	Yes	Moderate
Sharpton et al. (41)	Yes	Yes	No	Partial yes	Yes	Yes	Yes	Partial yes	Yes	No	Yes	Yes	Yes	Yes	Yes	Yes	Moderate
Loman et al. (40)	Yes	Yes	No	Partial yes	Yes	Yes	No	Yes	Yes	No	Yes	Yes	Yes	Yes	Yes	Yes	Moderate
Khan et al. (39)	Yes	Partial yes	No	Partial yes	Yes	Yes	No	Yes	Yes	No	Yes	Yes	Yes	Yes	No	Yes	Moderate
Tabrizi et al.	Yes	Partial yes	No	Yes	Yes	Yes	No	Yes	Yes	No	Yes	Yes	Yes	Yes	Yes	Yes	Moderate
Johan et al. (28)	Yes	Yes	No	Partial yes	Yes	Yes	No	Yes	Yes	Yes	Yes	Yes	Yes	Yes	Yes	Yes	Moderate
Beserra et al. (27)	Yes	Partial yes	No	Partial yes	Yes	Yes	No	Yes	Yes	No	Yes	Yes	Yes	Yes	Yes	Yes	Moderate
Arabi et al. (35)	Yes	Yes	Yes	Partial yes	Yes	Yes	Yes	Yes	Yes	No	Yes	Yes	Yes	Yes	Yes	No	High
Heshmati et al. (37)	Yes	Yes	Yes	Partial yes	Yes	Yes	Yes	Yes	Yes	No	Yes	Yes	Yes	Yes	Yes	Yes	High
Miao et al. (43)	No	Yes	Yes	Partial yes	Yes	Yes	Yes	Partial yes	Yes	Yes	Yes	Yes	Yes	No	Yes	Yes	High
Li et al. (58)	Yes	Yes	No	Partial yes	Yes	Yes	No	Yes	Yes	No	Yes	Yes	No	No	Yes	No	Moderate
Liu et al. (42)	Yes	Partial yes	No	Partial yes	Yes	Yes	No	Yes	Yes	No	Yes	Yes	Yes	Yes	Yes	Yes	Moderate
Hadi et al. (25)	No	Partial yes	No	Partial yes	No	Yes	No	Yes	Yes	No	Yes	Yes	Yes	Yes	Yes	Yes	Moderate
Hadi et al. (23)	Yes	Partial yes	No	Partial yes	Yes	Yes	No	Yes	Yes	No	Yes	Yes	Yes	Yes	Yes	Yes	Moderate
Hadi et al. (36)	No	Yes	Yes	Partial yes	Yes	Yes	Yes	Yes	Yes	No	Yes	Yes	Yes	Yes	No	Yes	Moderate

*1. Did the research questions and inclusion criteria for the review include the components of PICO? 2. Did the report of the review contain an explicit statement that the review methods were established prior to the conduct of the review and did the report justify any significant deviations from the protocol? 3. Did the review authors explain their selection of the study designs for inclusion in the review? 4. Did the review authors use a comprehensive literature search strategy? 5. Did the review authors perform study selection in duplicate? 6. Did the review authors perform data extraction in duplicate? 7. Did the review authors provide a list of excluded studies and justify the exclusions? 8. Did the review authors describe the included studies in adequate detail? 9. Did the review authors use a satisfactory technique for assessing the risk of bias (RoB) in individual studies that were included in the review? 10. Did the review authors report on the sources of funding for the studies included in the review? 11. If meta-analysis was performed, did the review authors use appropriate methods for statistical combination of results? 12. If meta-analysis was performed, did the review authors assess the potential impact of RoB in individual studies on the results of the meta-analysis or other evidence synthesis? 13. Did the review authors account for RoB in individual studies when interpreting/discussing the results of the review? 14. Did the review authors provide a satisfactory explanation for, and discussion of, any heterogeneity observed in the results of the review? 15. If they performed quantitative synthesis, did the review authors carry out an adequate investigation of publication bias (small study bias) and discuss its likely impact on the results of the review? 16. Did the review authors report any potential sources of conflict of interest, including any funding they received for conducting the review? Each question was answered with “Yes”, “Partial Yes” or “No”. When no meta-analysis was done, question 11, 12, and 15 were answered with “No meta-analysis conducted”.

### Synbiotic on BMI

**FIGURE 2:**
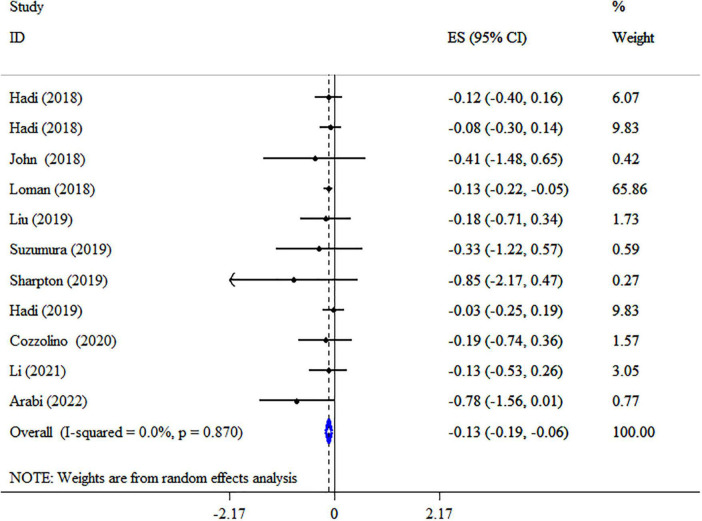
Forest plot with a mean difference and 95% confidence intervals (CIs) the impacts of synbiotic supplementation on BMI levels.

Synbiotic supplementation significantly decreased BMI (ES: −0.13 kg/m2; 95% CI: −0.19, −0.06, *p* < 0.001), according to a pooled analysis of 11 meta-analyses (Figure 2), without heterogeneity between-study (*I*^2^ = 0.0%, *p* = 0.870). Synbiotic supplementation in subjects with non-alcoholic fatty liver disease (NAFLD) with intervention >10 weeks, in studies with multi strains (*Bifidobacteria, Lactobacilli* plus *Streptococcus*) and a sample size of 200 individuals or over led to a remarkable reduction in BMI levels (Table 3). Also, the overall effects of synbiotics on BMI changed to non-significant after removing the Loman et al. (40) study by sensitivity analysis (ES: −0.12 kg/m^2^; 95% CI: −0.23, 0.001, *p* > 0.05). A significant small-study effect was detected using Begg’s (*p* = 0.002) unlike Egger’s (*p* = 0.076) tests. Visual inspection of the funnel plot revealed an uneven distribution of meta-analyses (Supplementary Figure 1). Thus, trim and fill methods was performed with 11 studies without imputed study (ES: −0.13 kg/m2; 95% CI: −0.19, −0.06, *p* < 0.05). The BMI quality of evidence was estimated as moderate performing the GRADE system (based on the indirectness) (Table 4).

**TABLE 3:** Subgroup analyses for the effects of synbiotic supplementation on obesity.

Synbiotic on BMI	Effect size, *n*	ES (95% CI)^1^	*P*-within^2^	*I*^2^ (%)^3^	P-heterogeneity^4^
Overall	11	−0.13 (−0.19, −0.06)	<0.001	0	0.870
**Age (year)**					
≤45	3	−0.07 (−0.26, 0.12)	0.478	0	0.787
>45	5	−0.13 (−0.21, −0.06)	0.001	0	0.553
NR	3	−0.21 (−0.57, 0.14)	0.240	0	0.550
**Intervention duration (week)**					
≤10	4	−0.08 (0.27, 0.12)	0.438	0	0.796
>10	7	0.13 (0.21, 0.06)	<0.001	0	0.675
**Study population**					
NAFLD	4	−0.13 (−0.21, −0.05)	0.001	0	0.712
Different diseases	2	−0.14 (−0.41, 0.13)	0.315	0	0.606
OW and OB	1	−0.33 (−1.23, 0.56)	0.470	–	–
PCOS	3	−0.07 (−0.25, 0.11)	0.459	0	0.819
Metabolic syndrome	1	−0.78 (−1.57, 0.01)	0.051	–	–
**Sample size**					
≤300	7	−0.13 (−0.29, 0.04)	0.128	0	0.682
>300	4	−0.13 (−0.20, −0.05)	0.001	0	0.721
**Dosage**					
10^9^–10^10^	4	−0.11 (−0.28, 0.06)	0.193	0	0.893
≥10^10^	4	−0.10 (−0.28, 0.07)	0.246	11.6	0.335
NR	3	−0.13 (−0.22, −0.05)	0.002	0	0.558
**Gender**					
Women	3	−0.07 (−0.25, 0.11)	0.459	0	0.819
Both	8	−0.14 (−0.21, −0.06)	<0.001	0	0.727
**Type of strains**					
Lacto + Bifido	4	−0.07 (−0.22, 0.07)	0.323	0.0	0.647
Lacto + Bifido + Strepto	7	−0.14 (−0.22, −0.06)	<0.001	0.0	0.531
**Synbiotic on body weight**					
Overall	8	−1.30 (−2.19, −0.41)	0.004	88.9	<0.001
**Age (years)**					
≤45	2	−0.13 (−0.59, 0.34)	0.594	0	0.507
>45	4	−2.24 (−3.72, −0.77)	0.003	87	<0.001
NR	2	−0.14 (−0.51, 0.22)	0.442	0	0.425
**Intervention duration (week)**					
≤10	3	−0.16 (−0.62, 0.29)	0.485	0	0.589
>10	5	−1.78 (−3.16, −0.39)	0.012	93	<0.001
**Study population**					
PCOS	3	−0.12 (−0.41, 0.17)	0.407	0	0.803
OW and OB	1	−1.24 (−2.58, 0.09)	0.069	–	–
Different diseases	2	−0.83 (−1.56, −0.10)	0.026	0	0.818
NAFLD	1	−2.98 (−3.78, −2.18)	<0.001	–	–
MetS	1	−4.38 (−6.21, 2.56)	<0.001	–	–
**Sample size**					
≤300	6	−0.94 (−1.76, −0.11)	0.026	78.6	<0.001
>300	2	−1.89 (−4.02, 0.25)	0.083	93.3	<0.001
Dosage ≤10^9^	1	−2.98 (−3.78, −2.18)	<0.001	–	–
10^9^–10^10^	5	−1.01 (−1.93, −0.09)	0.032	82.8	<0.001
>10^10^	2	−0.78 (−1.47, −0.08)	0.029	0	0.880
**Gender**					
Women	3	−0.12 (−0.41, 0.17)	0.407	0	0.803
Both	5	−2.07 (−3.38, −0.76)	0.002	83	<0.001
**Type of strains**					
Lacto + Bifido	4	−1.14 (−2.55, 0.27)	0.112	92.0	<0.001
Lacto + Bifido + Strepto	4	−1.60 (−3.37, 0.17)	0.077	86.5	<0.001
**Synbiotic on WC levels**					
Overall	7	−1.80 (−3.26, −0.34)	0.016	94.1	<0.001
**Age (years)**					
≤45	3	−2.01 (−4.64, 0.62)	0.135	97.6	<0.001
>45	2	−2.17 (−4.55, 0.21)	0.074	75.4	0.044
NR	2	−0.63 (−1.61, 0.35)	0.209	0	0.387
**Intervention duration (week)**					
≤10	4	−2.15 (−3.93,−0.37)	0.018	87.9	<0.001
>10	3	−1.33 (−3.34, 0.67)	0.193	88.2	<0.001
**Study population**					
NAFLD	2	−0.11 (−0.66, 0.45)	0.501	20	0.264
OW and OB	1	−2.07 (−3.11, −1.03)	<0.001	–	–
Different diseases	1	−3.39 (−5.07, −1.72)	<0.001	–	–
PCOS	2	−0.63 (−1.61, 0.35)	0.219	0	0.387
MetS	1	−4.04 (−4.99, −3.08)	<0.001	–	–
**Sample size**					
≤300	6	−1.75 (−3.43, −0.08)	0.040	94.3	<0.001
>300	1	−2.07 (−3.11, −1.03)	<0.001	–	–
Dosage (mg/day) <10^9^	1	−0.96 (−2.63, 0.70)	0.258	–	–
≥10^9^	4	−2.47 (−4.14, −0.80)	0.004	88.5	<0.001
NR	2	−0.32 (−1.68, 1.04)	0.646	33	0.222
**Gender**					
Women	2	−0.63 (−1.61, 0.35)	0.209	0	0.387
Both	5	−2.07 (−4.02, −0.12)	0.038	96	<0.001
**Type of strains**					
Lacto + Bifido	2	−1.71 (−2.73, −0.70)	<0.001	18.6	0.268
Lacto + Bifido + Strepto	5	−1.92 (−3.86, 0.03)	0.053	95.4	<0.001

ES, effect size; CI, confidence interval; L, Lactobacillus; B, Bifidobacterium; S, Streptococcus. ^1^Obtained from the Random-effects model, ^2^Refers to the mean (95% CI), ^3^Inconsistency, percentage of variation across studies due to heterogeneity, ^4^Obtained from the *Q*-test.

**TABLE 4:** Summary of findings and quality of evidence assessment using the GRADE approach.

Outcome measure	Summary of findings		Quality of evidence assessment (GRADE)					
	No of patients (meta-analysis)	Effect size* (95% CI)	Risk of bias^a^	Inconsistency^b^	Indirectness^c^	Imprecision^d^	Publication bias^e^	Quality of evidence^f^
**Anthropometric measures**								
BMI (kg/m^2^)	3,973 (11)	−0.13 (−0.19, −0.06)	Not serious	Not serious	Serious	Not serious	Not serious	Moderate
Body weight (kg)	2,593 (8)	−1.30 (−2.19, −0.41)	Not serious	Not serious	Serious	Not serious	Not serious	Moderate
WC (cm)	1,465 (7)	−1.80 (−3.26, −0.34)	Not serious	Not serious	Serious	Serious	Not serious	Low
**Lipid profile**								
LDL (mg/dl)	3,184 (10)	−2.81 (−3.90, −1.72)	Not serious	Not serious	Serious	Serious	Not serious	Low
HDL (mg/dl)	3,098 (10)	0.23 (−0.11, 0.56)	Not serious	Not serious	Serious	Not serious	Not serious	Moderate
TG (mg/dl)	3,393 (11)	−0.43 (−0.79, −0.07)	Not serious	Not serious	Serious	Not serious	Not serious	Moderate
TC (mg/dl)	3,258 (10)	−2.24 (−3.18, −1.30)	Not serious	Not serious	Serious	Not serious	Not serious	Moderate

BMI, body mass index; WC, waist circumference; LDL, low-density lipoprotein; HDL, high-density lipoprotein; TG, triglyceride; TC, total cholesterol. ^a^Risk of bias based on the AMSTAR results. ^b^Downgraded if there was a substantial unexplained heterogeneity (*I*^2^ > 50%, *P* < 0.10) that was unexplained by meta-regression or subgroup analyses. ^c^Downgraded if there were factors present relating to the participants, interventions, or outcomes that limited the generalizability of the results. ^d^Downgraded if the 95% confidence interval (95% CI) crossed the minimally important difference (MID) for benefit or harm. MIDs used for each outcome were: 3.87 mg/dl for LDL, HDL, and TC, 8.86 mg/dl for TG, 0.2 kg/m^2^ for BMI, and 2 cm for WC, 5–10% for body weight Viguiliouk et al. (77). ^e^Downgraded if there was an evidence of publication bias using funnel plot. ^f^Since all included studies were meta-analyses, the certainty of the evidence was graded as high for all outcomes by default and then downgraded based on prespecified criteria. Quality was graded as high, moderate, low, very low.

### Synbiotics on BW

**FIGURE 3:**
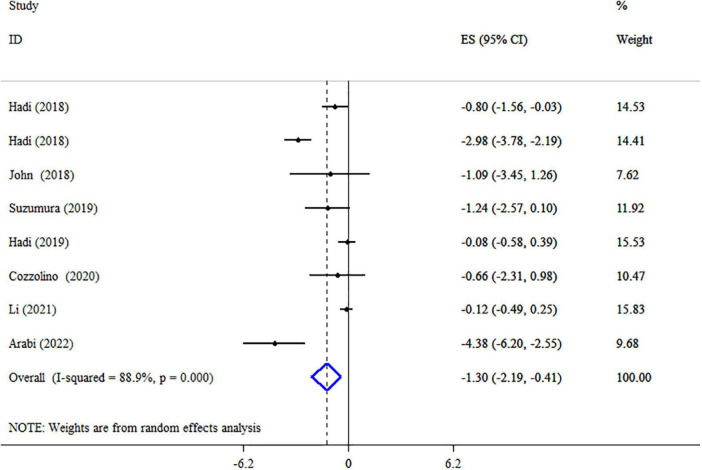
Forest plot detailing mean difference and 95% confidence intervals (CIs), the impacts of synbiotic supplementation on body weight.

A pooled analysis of eight meta-analyses revealed that synbiotic supplementation reduced BW significantly (ES: −1.30 kg; 95% CI: −2.19, −0.41, *p* = 0.004) (Figure 3). However, there was high heterogeneity between studies (*I*^2^ = 88.9%, *p* < 0.001), which was decreased after subgroup analysis based on the dosage of synbiotic, duration of intervention, mean age, gender, and health condition (Table 3). Subgroup analysis demonstrated that synbiotic supplementation with a dosage of ≤10^9^ CFU and subjects with a mean age of >45 years in the duration of intervention >10 weeks contributes to a more effective in reducing BW (Table 3). The sensitivity analysis showed that calculated overall ESs for BW alterations were not significantly changed after omitting each study. Based on Begg’s test, no evidence of publication bias was found (*p* = 0.266). The quality of evidence related to BW was downgraded to moderate due to serious limitations in indirectness (Table 4).

### Synbiotic on WC

**FIGURE 4:**
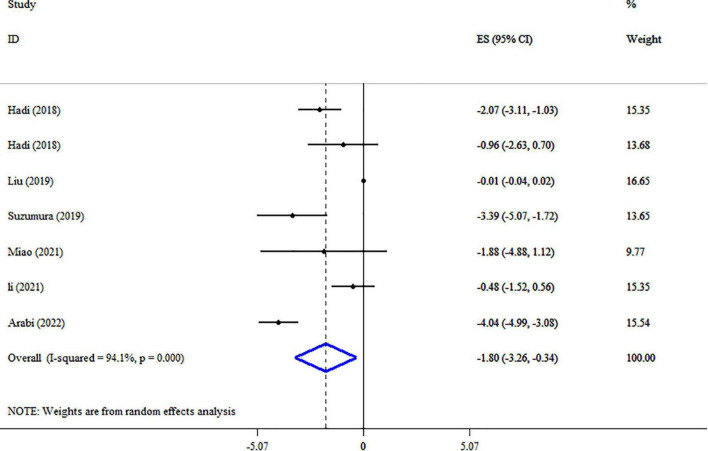
Forest plot detailing mean difference and 95% confidence intervals (CIs), the impacts of synbiotic supplementation on WC.

A pooled analysis of seven studies including 1,465 participants indicated that synbiotic supplementation causes a significant reduction in WC (ES: −1.80 cm; 95% CI: −3.26, −0.34, *p* = 0.016) (Figure 4). There was a significant between-study heterogeneity (*I*^2^ = 94.1%, *p* < 0.001). Dosage, duration of intervention, strains of bacteria, and health conditions were detected as sources of heterogeneity in the subgroup analysis (Table 3). Synbiotic supplementation in subjects aged >45 years, duration of intervention ≤10 weeks, and intervention doses of ≥10^9^ CFU led to a substantial reduction of WC levels. Also, we found a significant reduction in WC levels when used in studies with *Bifidobacteria* plus *Lactobacilli* strains (Table 3). Performing sensitivity analysis, there was no significant change when one particular study was removed. Begg’s test indicated no significant publication bias (*p* = 0.649). Based on the GRADE approach, the overall quality of the evidence for WC was considered low due to serious indirectness and imprecision (Table 4).

### Synbiotic on LDL-c

**FIGURE 5:**
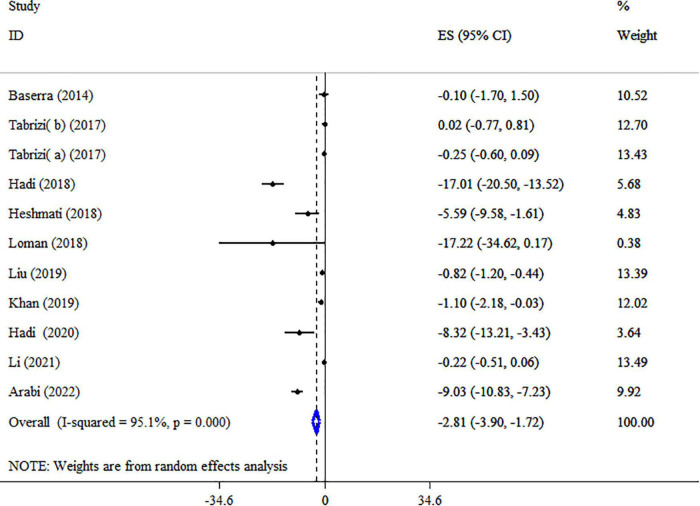
Forest plot detailing mean difference and 95% confidence intervals (CIs), the impacts of synbiotic supplementation on LDL-C levels.

Synbiotic supplementation meaningfully reduced LDL-C level based on the 10 meta-analyses with 11 ESs (ES: −2.81 mg/dl; 95% CI: −3.90, −1.72, *p* < 0.001) (Figure 5). Significant between-study heterogeneity was detected (*I*^2^ = 95.1%, *p* < 0.001). The dosage of synbiotics, mean age, and health condition were identified as sources of heterogeneity after subgroup analysis (Table 5). Subgroup analysis indicated that synbiotic supplementation in subjects with NAFLD with a dosage of 10^9^–10^10^ CFU, intervention duration of ≥15-weeks, and type of ES weighted mean difference (WMD) contributes to a greater impact in the lowering LDL-C concentrations (Table 5). The following analysis indicated there was a significant reduction in LDL levels when using *Bifidobacteria* plus *Lactobacilli* strains (Table 5). A sensitivity analysis found that no special study affected the overall ES. Egger’s and Begg’s tests identified a small-study effect (*p* = 0.018 and *p* = 0.029, respectively), also a visual inspection of the funnel plot revealed the presence of publication bias (Supplementary Figure 2). Therefore, Trim and fill analysis was carried out with 11 studies (ES: −2.81 mg/dl; 95% CI: −3.90, −1.72, *p* < 0.05). As shown in Table 4, the LDL-C quality of evidence was rated as low using the GRADE system (based on the indirectness and imprecision).

**TABLE 5:** Subgroup analyses for the effects of synbiotic supplementation on lipid profile.

	Effect size, *n*	ES (95% CI)^1^	*P*-within^2^	*I*^2^ (%)^3^	*P*-heterogeneity^4^
**Synbiotic on TG levels**					
Overall	12	−0.43 (−0.79, −0.07)	0.019	78	<0.001
**Age (years)**					
<50	7	−0.45 (−0.97, 0.07)	0.088	68.8	0.004
≥50	4	−0.71 (−1.63, 0.22)	0.134	89.5	<0.001
NR	1	−0.14 (−0.47, 0.2)	0.413	–	–
**Intervention duration (week)**					
<15	7	−0.36 (−0.70, −0.01)	0.045	70.8	0.002
≥15	5	−0.90 (−2.10, 0.29)	0.138	86.3	<0.001
**Study population**					
PCOS	3	−10.94 (−26.38, 4.5)	0.165	84	0.002
NAFLD	4	−0.62 (−1.6, 0.35)	0.212	84.5	<0.001
T2DM	2	−0.37 (−0.56, −0.18)	<0.001	0	0.625
OW and OB	1	−0.43 (−0.7, −0.16)	0.002	–	–
Other	1	−14.30 (−25.32, −3.28)	0.011	–	–
MetS	1	−20.30 (−32.72, −7.88)	0.001	–	–
**Sample size**					
≤200	6	−0.35 (−0.82, 0.13)	0.154	65.3	0.013
>200	6	−0.56 (−1.16, 0.05)	0.071	85.7	<0.001
**Dosage**					
≤10^9^	1	−0.49 (−0.87, −0.11)	0.011	–	–
10^9^–10^10^	6	−1.16 (−2.48, 0.16)	0.085	88.3	<0.001
>10^10^	1	−14.30 (−25.32, −3.28)	0.011	–	–
NR	4	−0.36 (−0.52, −0.2)	<0.001	0	0.923
**Gender**					
Women	3	−10.94 (−26.38, 4.5)	0.165	84	0.002
Both	9	−0.45 (−0.82, −0.09)	0.016	77.6	<0.001
**Type of effect size**					
WMD	5	−19.15 (−24.62, −13.69)	<0.001	0	0.778
SMD	6	−0.36 (−0.48, −0.24)	<0.001	0	0.759
NR	1	−11.11 (−55.9, 33.69)	0.627	–	–
**Strains of bacteria**					
Lacto	1	−0.40 (−0.60, −0.16)	<0.001	–	–
Lacto + Bifido	6	−0.83 (−1.94, 0.27)	0.140	84.2	<0.001
Lacto + Bifido + Strepto	5	0.39 (0.93, 0.15)	0.153	77.8	<0.001
**Synbiotic on TC levels**					
Overall	11	−2.24 (−3.18, −1.30)	<0.001	94.5	<0.001
**Sample size**					
≤200	6	−2.46 (−3.86, −1.07)	0.001	96.3	<0.001
>200	5	−2.48 (−4.15, −0.81)	0.004	90.9	<0.001
**Type of effect size**					
WMD	4	−9.35 (−15.06, −3.64)	0.001	68.2	0.024
SMD	6	−0.34 (−0.50, −0.18)	<0.001	0	0.535
NR	1	−14.89 (−17.34, −12.44)	<0.001	–	–
**Gender**					
Both	9	−3.08 (−4.32, −1.85)	<0.001	95.6	<0.001
Women	2	−0.28 (−0.56, 0.00)	0.054	0	0.990
**Age**					
≤50	6	−4.19 (−6.49, −1.89)	<0.001	96.5	<0.001
>50	4	−1.54 (−3.05, −0.02)	0.047	90.6	<0.001
NR	1	−0.28 (−0.56, 0.01)	0.054	–	–
**Health**					
NAFLD	4	−7.58 (−12.66, −2.50)	0.003	98	<0.001
T2DM	2	−0.33 (−0.70, 0.05)	0.085	0	0.656
PCOS	2	−0.28 (−0.56, 0.00)	0.054	0	0.990
OW and OB	1	−0.20 (−0.52, 0.12)	0.228	–	–
Other	1	−10.17 (−15.74, −4.60)	<0.001	–	–
MetS	1	−7.81 (−12.60, −3.03)	0.001	–	–
**Dose**					
≤10^9^	3	−0.40 (−0.76, −0.05)	0.027	0.1	0.368
10^9^–10^10^	5	−0.99 (−2.09, 0.12)	0.080	87.4	<0.001
>10^10^	1	−10.17 (−15.74, −4.60)	<0.001	–	–
NR	2	−7.66 (−21.73, 6.42)	0.286	99.2	<0.001
**Duration**					
≤15	6	−0.32 (−0.69, 0.05)	0.089	60.3	0.027
>15	5	−7.62 (−12.21, −3.03)	<0.001	97.5	<0.001
**Strains of bacteria**					
Lacto	1	−0.25 (−0.75, 0.25)	0.327	–	–
Lacto + Bifido	5	−5.66 (−8.60, 2.71)	<0.001	97.5	<0.001
Lacto + Bifido + Strepto	5	−1.00 (−1.84, −0.15)	0.020	83.2	<0.001
**Synbiotic on LDL levels**					
Overall	11	−2.81 (−3.90, −1.72)	<0.001	95.1	<0.001
**Sample size**					
≤200	7	−0.56 (−1.01, −0.10)	0.017	68.1	0.004
>200	4	−8.48 (−16.03, −0.93)	0.028	98.1	<0.001
**Type of effect size**					
WMD	4	−10.06 (−14.55, −5.58)	<0.001	86.2	<0.001
SMD	6	−0.40 (−0.70, −0.10)	0.008	48.6	0.083
NR	1	−17.22 (−34.61, 0.17)	0.052	–	–
**Gender**					
Both	9	−3.52 (−5.04, −2.00)	<0.001	95.8	<0.001
Women	2	−2.52 (−7.73, 2.69)	0.343	85.6	0.008
**Age**					
<50	6	−1.18 (−2.08, −0.28)	0.010	80.1	<0.001
≥50	4	−6.32 (−12.13, −0.51)	0.033	98.1	<0.001
NR	1	−0.22 (−0.50, 0.07)	0.130	–	–
**Health**					
NAFLD	4	−6.14 (−10.22, −2.06)	0.003	96.5	<0.001
Diabetes	2	−0.21 (−0.52, 0.11)	0.200	0	0.539
PCOS	2	−2.52 (−7.73, 2.69)	0.343	85.6	0.008
OW and OB	1	−0.10 (−1.70, 1.50)	0.903	–	–
Other	1	−8.32 (−13.21, −3.43)	<0.001	–	–
MetS	1	−9.03 (−10.83, −7.23)	<0.001	–	–
**Dose**					
≤10^9^	2	−0.48 (−1.57, 0.61)	0.390	63.1	0.100
10^9^–10^10^	5	−6.20 (−11.04, −1.37)	0.012	97.8	<0.001
NR	3	−0.56 (−1.25, 0.13)	0.114	75.9	0.016
>10^10^	1	−8.32 (−13.21, −3.43)	<0.001	–	–
**Duration**					
<15	5	−0.45 (−1.10, 0.21)	0.182	77.6	0.001
≥15	6	−5.58 (−8.92, −2.23)	<0.001	96.9	<0.001
**Strains of bacteria**					
Lacto	1	−0.02 (−0.77, 0.81)	0.096	–	–
Lacto + Bifido	5	−6.12 (−10.94, −1.31)	0.013	95.9	<0.001
Lacto + Bifido + Strepto	5	−2.84 (−4.38, −1.29)	<0.001	96.1	<0.001
**Synbiotic on HDL levels**					
Overall	11	0.23 (−0.11, 0.56)	0.193	45.2	0.051
**Sample size**					
≤200	7	0.14 (−0.14, 0.343)	0.325	27.3	0.220
>200	4	0.94 (−0.44, 2.33)	0.182	69.0	0.022
**Type of effect size**					
WMD	4	1.59 (0.79, 2.39)	<0.001	0	0.882
SMD	6	0.07 (−0.14, 0.29)	0.490	0	0.460
NR	1	1.08 (−6.68, 8.85)	0.785	–	–
**Gender**					
Both	9	0.15 (−0.21, 0.51)	0.418	39.6	0.103
Women	2	0.75 (−0.75, 2.25)	0.328	78.1	0.033
**Age**					
≤50	6	0.34 (−0.22, 0.91)	0.235	54.9	0.049
>50	4	0.31 (−0.52, 1.14)	0.467	56.2	0.077
NR	1	0.09 (−0.47, 0.66)	0.755	–	–
**Health**					
NAFLD	4	0.06 (−0.43, 0.56)	0.800	23.8	0.268
Diabetes	2	−0.26 (−0.88, 0.35)	0.397	0	0.547
PCOS	2	0.75 (−0.75, 2.25)	0.328	78.1	0.033
OW and OB	1	0.15 (−0.28, 0.57)	0.489	–	–
Other	1	1.30 (0.03, 2.57)	0.044	–	–
MetS	1	2.32 (0.19, 4.44)	0.032	–	–
**Dose**					
≤10^9^	3	0.08 (−0.29, 0.45)	0.670	15.6	0.306
10^9^–10^10^	5	0.61 (−0.05, 1.27)	0.070	56.8	0.055
>10^10^	1	1.30 (0.03, 2.57)	0.044	–	–
NR	2	−0.43 (−1.13, 0.27)	0.233	0	0.702
**Duration**					
<15	6	0.26 (–0.20, 0.72)	0.272	51.6	0.067
≥15	5	0.29 (–0.45, 1.02)	0.446	49.6	0.094
**Strains of bacteria**					
Lacto	1	−0.43 (−1.25, 0.38)	0.301	–	–
Lacto + Bifido	4	0.14 (−0.24, 0.53)	0.461	0.0	0.340
Lacto + Bifido + Strepto	6	0.47 (−0.09, 1.03)	0.100	65.8	0.051

ES, effect size; CI, confidence interval; L, lactobacillus; B, Bifidobacterium; S, streptococcus. ^1^Obtained from the Random-effects model, ^2^Refers to the mean (95% CI), ^3^Inconsistency, percentage of variation across studies due to heterogeneity, ^4^Obtained from the *Q*-test.

### Synbiotic on TC

**FIGURE 6:**
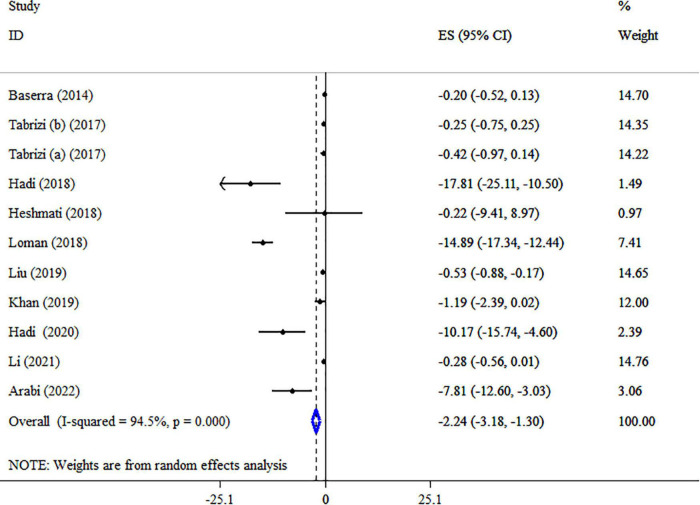
Forest plot detailing mean difference and 95% confidence intervals (CIs), the impacts of synbiotic supplementation on TC levels.

The effect of synbiotics on TC level was reported in 10 studies with 11 ESs (Figure 6). Our analysis revealed a significant reduction in TC levels after synbiotic administration (*ES* = −2.24 mg/dl, 95% CI: −3.18, −1.30, *p* < 0.001), with high heterogeneity between-study (*I*^2^ = 94.5%, *p* < 0.001), which was decreased after subgroup analysis based on the dosage of synbiotic, duration of intervention, strains of bacteria, type of ESs and health condition. Subgroup analysis showed that the impacts of synbiotic reduction on TC were more robust in studies with participants with NAFLD, age ≤50 years, the sample size of ≤200, type of ES (WMD), and multi strains of bacteria (*lactobacillus, bifidobacterium*, plus *streptococcus*) than the entire sample (Table 5). Sensitivity analysis for TC concentrations did not show evidence of sensitivity. A considerable small-study effect was indicated using Egger’s and Begg’s tests (*p* = 0.015 and 0.029, respectively). Also, a visual inspection of the funnel plot found a significant publication bias among included studies (Supplementary Figure 3). Therefore, trim and fill analysis was conducted with 11 studies (no imputed studies) (*ES* = −2.24 mg/dl, 95% CI: −3.18, −1.30, *p* < 0.05). The quality of evidence related to TC was downgraded to moderate due to serious limitations in indirectness (Table 4).

### Synbiotic on TG

**FIGURE 7:**
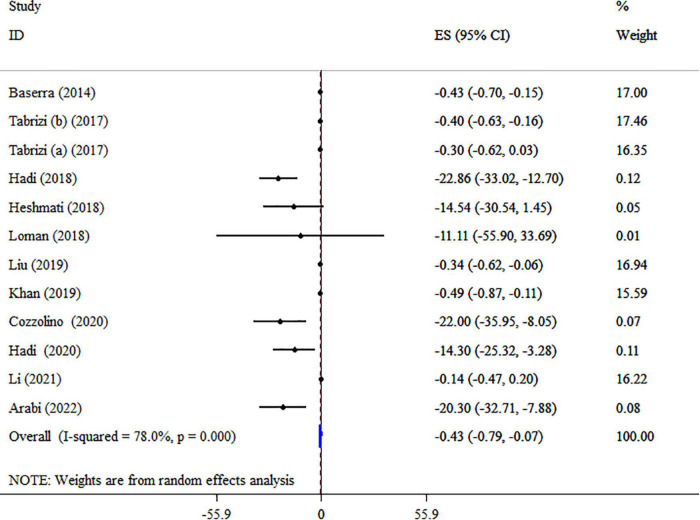
Forest plot detailing mean difference and 95% confidence intervals (CIs), the impacts of synbiotic supplementation on TG levels.

Eleven meta-analyses with 12 ESs, including 3,393 participants, have evaluated the effect of synbiotics on TG levels (Figure 7). The analysis indicated a significant decrease of TG by synbiotic supplementation (ES: −0.43 mg/dl; 95% CI: −0.79, −0.07, *p* = 0.019). However, significant heterogeneity was detected among studies (*I*^2^ = 78.0%, p < 0.001). The dosage of synbiotics, sample size, health status, and type of ES was identified as sources of heterogeneity in the subgroup analysis (Table 5). The effects of the synbiotics on TG in the type of ES (WMD), and participants with T2DM were more robust than the entire sample (Table 5). No evidence of the effect of a single study on the overall ES was detected using sensitivity analysis. A substantial small-study effect was shown using Egger’s unlike Begg’s tests (*p* < 0.001 and 0.244, respectively). After a visual inspection of the funnel plot, publication bias was observed (Supplementary Figure 4). Following trim and fill analysis, the overall ES did not alter (ES: −0.43 mg/dl; 95% CI: −0.79, −0.07, *p* < 0.05). According to the GRADE approach, TG was considered to have a moderate quality of evidence due to indirectness (Table 4).

### Synbiotic on HDL-c

**FIGURE 8:**
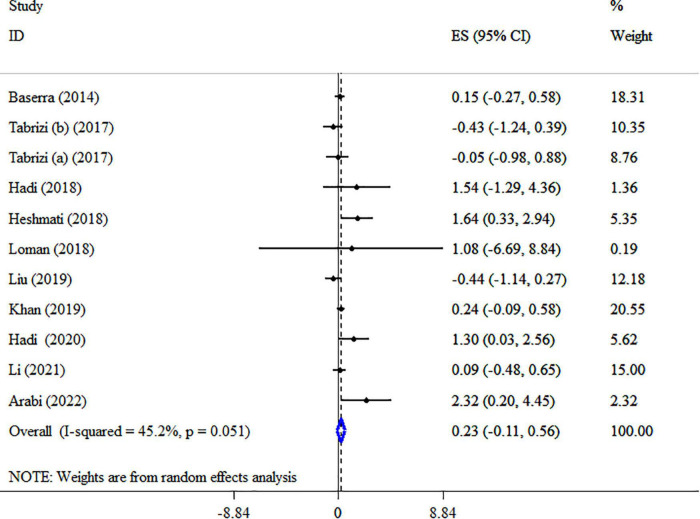
Forest plot detailing mean difference and 95% confidence intervals (CIs), the impacts of synbiotic supplementation on HDL-C levels.

Overall result from 10 meta-analyses containing 11 total ESs did not reveal significant alterations in HDL-C (ES: 0.23 mg/dl; 95% CI: −0.11, 0.56, *p* = 0.193; *I*^2^ = 45.2%, *p* = 0.051) (Figure 8). Also, in studies with type ES (WMD), a significant increase was observed in HDL-C levels (Table 5). Performing sensitivity analysis, there was no significant change when one single study was removed. There were no small-study effects using Egger’s and Begg’s tests (*P* = 0.189 and 0.350, respectively). In addition, a visual inspection of the funnel plot revealed asymmetry (Supplementary Figure 5). Therefore, the trim and fill test was carried out, and with imputing three fictitious studies, the result was still non-significance (ES: 0.08 mg/dl, 95% CI: −0.3, 0.5, *p* > 0.05). The HDL-C quality of evidence was rated as moderate using the GRADE system (based on the indirectness) (Table 4).

## Discussion

The current umbrella review and meta-analysis summarized the results of 17 meta-analyses, involving a total of 7,772 participants, and demonstrated that synbiotic supplementation could lead to a significant decrease in TG, TC, LDL-C, BW, BMI, and WC, nevertheless, no meaningful change was observed in HDL-C levels.

Stratifying the studies in different subgroups demonstrates different features of the effects of supplementation with synbiotics on lipid profile and anthropometric parameters and could help with gaining a conclusive result. For instance, stratifying the studies by the mean age of participants revealed that synbiotics had stronger effects on anthropometric parameters and LDL-C among older individuals (>45) in comparison with younger people. In contrast, the supplementation of synbiotics had a weaker impact on TC among individuals ≥50 years old in comparison with young adults, which is in line with a previous umbrella meta-analysis (46). The inconsistent findings might be due to the menopausal status of women. Menopause could affect lipid profile and the metabolism of lipoprotein namely LDL-C since the production of estrogen from ovaries reduces after menopause (47). According to the findings of a recent meta-analysis, in post-menopausal individuals, the levels of TG, TC, and LDL-C were higher than in women in pre-menopausal status. Thus, gender could be a potential factor in changing the final result, hence, most studies were conducted on both sex. Of note, almost all studies conducted on both genders showed more considerable effects of supplementation with synbiotics on lipid profile and anthropometric parameters than studies performed on women alone. Moreover, the present study shown that the impacts of synbiotic on lipid profile, BW, and WC were in a dose-dependent manner.

It should be mentioned that different health conditions could also affect the efficacy of synbiotic supplementation on the studied outcomes substantially. As an example, the administration of synbiotics had more promising impacts on BMI, body weight, TC, and LDL-C in NAFLD patients. On the other hand, among overweight and obese participants, synbiotics had a more substantial lowering effect on WC. Regarding TG levels, type 2 diabetic patients demonstrated more amending change following synbiotic administration. The lipid profile-lowering property of synbiotics could be due to its effect on insulin sensitivity, especially in NAFLD and diabetic patients (48, 49). As a large sample size reflects the higher power of the studies, studies with large sample sizes (more than 200 in lipid profile and 300 in anthropometric indices) exhibit more considerable results than studies with small ones. Nonetheless, synbiotic supplementation showed more promising effects on TC and BW with small sample sizes (≤200 and ≤300, respectively). Therefore, it could be concluded that sample size is not an important effective factor in the association between synbiotics and the aforementioned outcomes.

Another possible factor, which might affect the overall findings, is treatment duration. Short-term administration of synbiotics (≤10 weeks for anthropometric indices, ≤15 weeks for TC, and <15 weeks for LDL-C) resulted in smaller effects on BMI, body weight, TC, and LDL-C compared with long-term supplementation. The synbiotic effect on TG was not time-dependent. Also, WC was reduced significantly only in the subgroup of ≤10-week.

It needs to be mentioned that, in addition to the aforementioned factors like sample size and intervention duration, the different types of synbiotics could be of great importance. For instance, after subgroup analysis for strains of bacteria, synbiotic supplementation with two strains of Lactobacillus and Bifidobacterium significantly decreased WC by 1.71 cm and LDL-C by 6.12 mg/dl. Also, synbiotic supplementation with three strains of Lactobacillus, Bifidobacterium, and Streptococcus significantly decreased BMI by 0.14 kg/m^2^, TC by 1.00 mg/dl, and TG by 0.39 mg/dl. Numerous animal and human studies have demonstrated the beneficial effects of different strains of synbiotics, particularly those belonging to lactic acid bacteria and bifidobacteria, on lipid profile and anthropometric indices. In a study on healthy rats, Hosseinfard et al. (50) revealed that Lactobacillus. Plantarum significantly amended lipid profile levels namely TG, LDL-C, HDL-C, and TC. In another study, the weight-lowering property of *L. Plantarum* and *L. gasseri* on obese humans and animals was approved (51). An additional human study confirmed the effectiveness of special strains of synbiotics such as *L. acidophilus, L. casei*, and *Bifidobacterium bifidum* in reducing the levels of lipid profile (52). Other species of lactic acid bacteria and *bifidobacteria*, which could amend lipid profile levels in humans are as follows: *Bifidobacterium animalis* (53), *Bifidobacterium infantis, Bifidobacterium breve*, etc. (54), *and L. acidophilus, L. casei; L. lactis*, etc (55). The mechanism of action of different strains of synbiotic is through various ways indicating that weight-lowering and lipid profile-lowering characteristic of synbiotics is strain-specific. For example, in a study by Nabhani et al. (56), a combination of several strains namely *L. fermentum, L. plantarum, L. acidophilus, L. Gasseri* did not lead to a significant decrease in lipid profiles. In contrast, another study showed the administration of *L. acidophilus, L. casei*, and *B. bifidum* resulted in substantial alterations in LDL-C and HDL-C levels (57). Meanwhile, it has been demonstrated that the *L. casei* has a stronger effect on attenuating obesity than B. animalis VKB and VKL strains (58).

Because nearly all included studies had a low risk of bias, the findings of the present study could be dependable. In one study conducted by Hadi et al. (15), the population of the included studies was patients who had different health conditions, which might affect the generalizability of their results. It should be noted that the population of the included studies in the present research, in most cases, were patients with some health conditions namely NAFLD, MetS, overweight, and obesity, who were more likely to suffer from hyperlipidemia. Therefore, the findings of the current umbrella meta-analysis could be generalizable. Of note, regarding the assessed outcomes, we did not find any considerable unexplained heterogeneity, thus, the results of the study have consistency.

The mechanism of anti-obesity properties of synbiotics has been examined in several studies. The following mechanisms have been proposed: modulating lipid absorption and excretion (59), the activation of FXR receptor leading to a decrease in gluconeogenesis and mediating insulin production and glucose detonation (60, 61), attenuating hunger *via* elevating the levels of glucagon-like-peptides (GLP-1 and GLP-2) (62), and inhibiting lipogenesis and stimulating B-oxidation of fatty acids *via* modulating the expression of PPAR-a, ACAT, FAS, and SREBP-1 (63, 64). Although the exact mechanism of the action of synbiotics in modulating lipid profiles has remained unclear, numerous studies have suggested several mechanisms. It has been suggested that synbiotics could lower TC levels *via* cholesterol assimilation or deconjugation of bile salts (65–69). Besides, synbiotics could increase HDL-C levels through increased bile salt hydrolase activity (70). Probiotics might affect the levels of lipid profile *via* alleviating the activation of Toll-like receptor-4 (TLR-4) and the production of inflammatory cytokines, which consequently leads to a reduction in lipid profile (71). On the other hand, probiotics can modulate cholesterol metabolism *via* producing short-chain fatty acids (SCFAs) resulting in the prevention of the activation of hydroxymethyl glutaryl CoA reductase (HMG-CoA reductase), a rate-limiting enzyme in the cholesterol synthesis pathway (72). Other possible mechanisms regarding the cholesterol-lowering property of synbiotics include the following: conversion of cholesterol into coprostanol (73) and integrating cholesterol in cellular membranes (74). Besides, synbiotics could alleviate lipid profile levels *via* decreasing the secretion of VLDL, insulin resistance, inflammation, and *de novo* lipogenesis mediated by carbohydrate-responsive element-binding protein (ChREBP)/sterol regulatory element-binding protein (SREBP). Synbiotics also could reduce the accumulation of TG in adipose tissues and the liver by increasing the serum levels of the fasting-induced adipose factor (FIAF) following the inhibition of hepatic lipogenic enzymes mediated by ChREBP and SREBP-1c (75). On the other hand, increased levels of FIAF prohibit the release of TG from VLDL and chylomicrons *via* inhibiting endothelial lipoprotein lipase (LPS). Moreover, the increased levels of GLP-1 restrain the activity of gut lipases, which in turn leads to a decrease in TG absorption from the intestine (76).

### Limitations

On the whole, in the present umbrella meta-analysis, all of the performed meta-analyses of RCTs, which addressed the impacts of synbiotics on lipid profile and anthropometric indices were included. The evaluation of possible biases was carried out. The results were assessed based on different subgroups. However, one of the important and instinctive limitations of the umbrella meta-analysis is that some similar RCTs from various meta-analyses would be re-analyzed in the umbrella meta-analysis, and as a result, these similar RCTs experienced an increased weight in the analysis; therefore, actual results may be even weaker than those acquired.

## Conclusion

According to the results of the current study, synbiotic supplementation can attenuate TG, LDL-C, and TC levels as well as BW, BMI, and WC. Therefore, synbiotics might be a therapeutic option for obesity and its related disorders. However, it must be noted that several factors namely the intervention duration, synbiotic strains, and different health conditions could vary the beneficial effects of synbiotics.

## Data availability statement

The original contributions presented in this study are included in this article/Supplementary material, further inquiries can be directed to the corresponding author.

## Author contributions

VM, MV, and MMA wrote the original manuscript and contributed to the conception of the manuscript. SSA and VM contributed to data collection, analysis, and manuscript drafting. MV and AK provided the advice and consultation. PD and VM contributed to the final revision of the manuscript. All authors read and approved the final manuscript.
