# Supplementary material for: Effects of synbiotics supplementation on anthropometric and lipid profile parameters: Finding from an umbrella meta-analysis
Source: Front Nutr. 2023 Feb 23;10:1121541. doi: 10.3389/fnut.2023.1121541 (PMC9995782; doi:10.3389/fnut.2023.1121541)
Supplement: Supplementary file 1 [file Data_Sheet_1.docx]

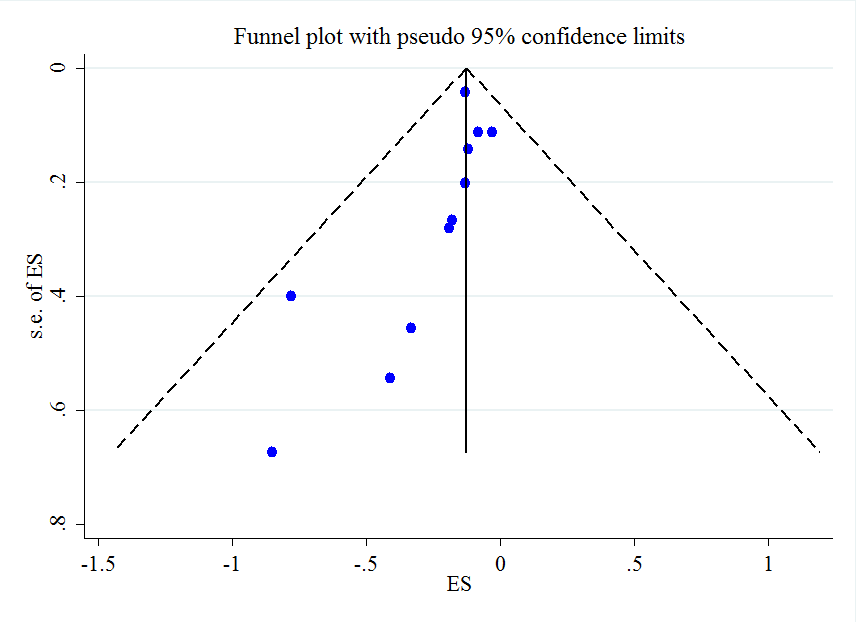
BMI

Funnel plots with a mean difference and 95% confidence intervals (CIs) the impacts of synbiotic supplementation on lipid profile and anthropometric parameters.


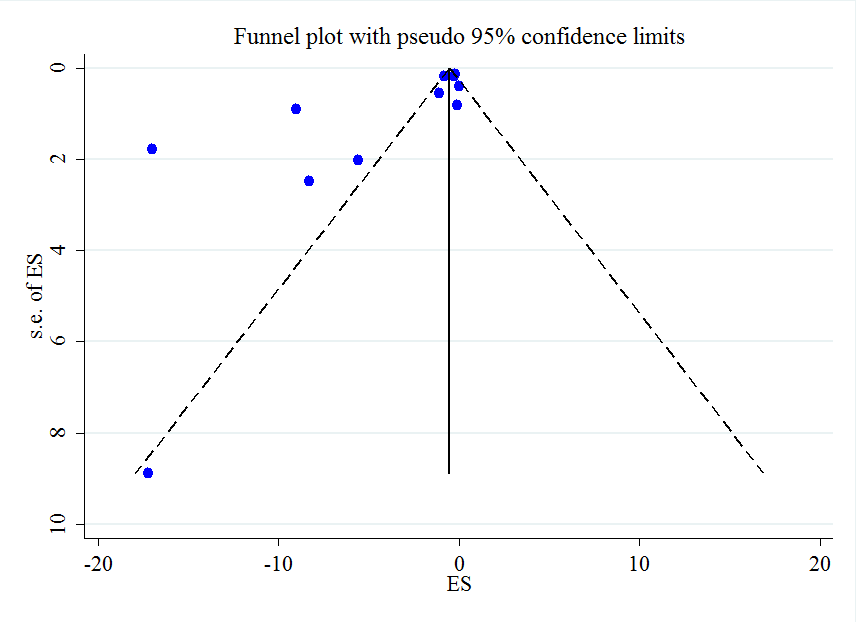
LDL-C


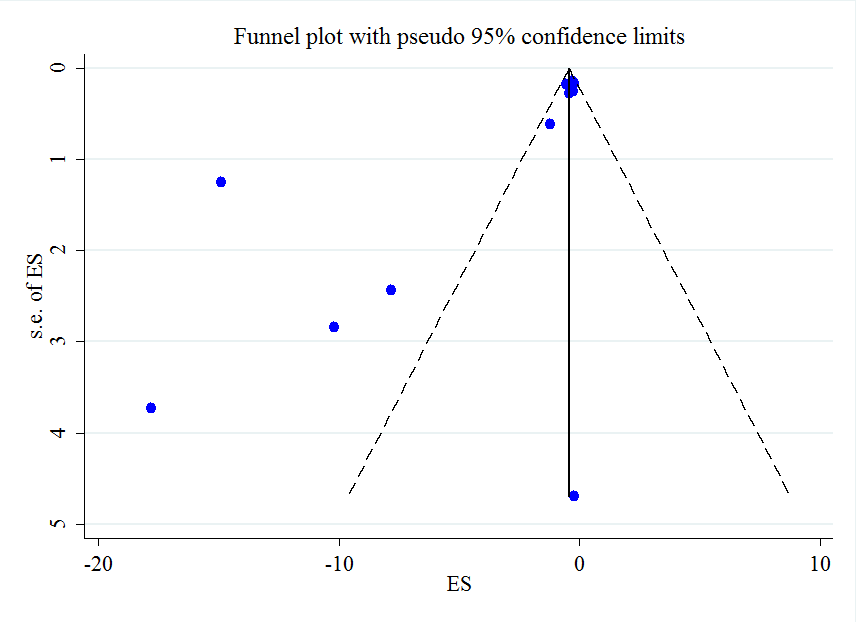
TC


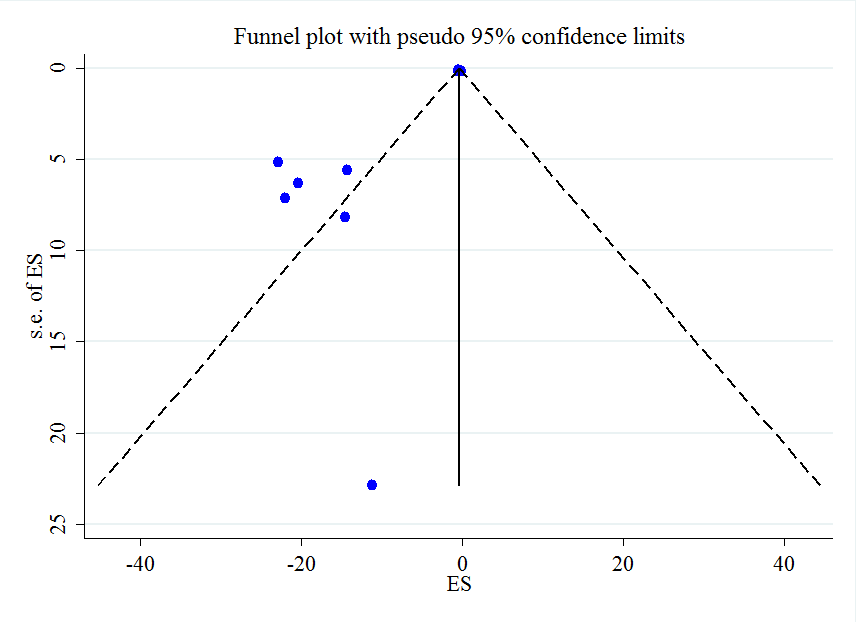
TG


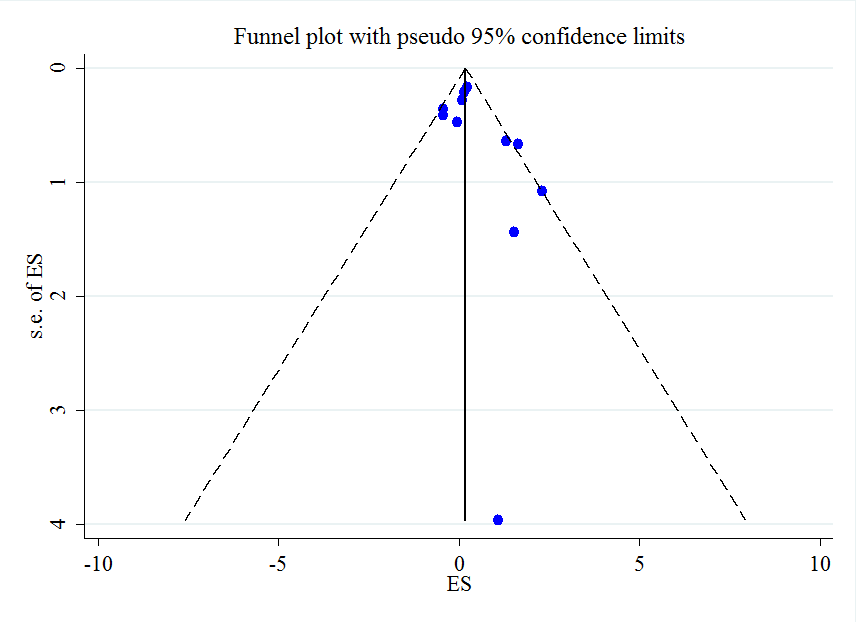
HDL-C
